# The role of the cerebellum in reconstructing social action sequences: a pilot study

**DOI:** 10.1093/scan/nsz032

**Published:** 2019-04-30

**Authors:** Frank Van Overwalle, Sarah De Coninck, Elien Heleven, Gaetano Perrotta, Nordeyn Oulad Ben Taib, Mario Manto, Peter Mariën

**Affiliations:** 1 Vrije Universiteit Brussel, Department of Psychology, Brussels, Belgium; 2 Department of Inclusive Society, University College Leuven-Limburg, Diepenbeek, Belgium; 3 Neurology Department, Hôpital Erasme, Université Libre de Bruxelles, Brussels, Belgium; 4 University Medical Center, Saint-Pierre, Brussels, Belgium; 5 Neurology Deparment, University Medical Center, Charleroi, Belgium and Neuroscience Department, Université de Mons, Mons, Belgium

**Keywords:** cerebellum, theory of mind, social mentalizing

## Abstract

Recent research has revealed that the cerebellum plays a critical role in social reasoning and in particular in understanding false beliefs and making trait attributions. One hypothesis is that the cerebellum is responsible for the understanding of sequences of motions and actions, which may be a prerequisite for social understanding. To investigate the role of action sequencing in mentalizing, we tested patients with generalized cerebellar degenerative lesions on tests of social understanding and compared their performance with matched healthy volunteers. The tests involved understanding violations of social norms making trait and causal attributions on the basis of short behavioral sentences and generating the correct chronological order of social actions depicted in cartoons (picture sequencing task). Cerebellar patients showed clear deficits only on the picture sequencing task when generating the correct order of cartoons depicting false belief stories and showed at or close to normal performance for mechanical stories and overlearned social scripts. In addition, they performed marginally worse on trait attributions inferred from verbal behavioral descriptions. We conclude that inferring the mental state of others through understanding the correct sequences of their actions requires the support of the cerebellum.

When maneuvering through the social environment, it is crucial to understand the mind of other persons. The capacity to capture other persons’ intentions, beliefs, emotions and personality traits based on behavioral descriptions is termed `mind reading’ or `mentalizing’. During the past decade, social neuroscience has made great progress in understanding the neural correlates of mentalizing. However, social research has focused predominantly on the role of the cerebrum and its cortical areas subserving mentalizing, collectively termed the mentalizing network (for reviews, see Van Overwalle, 2009; Schurz *et al.*, 2014; Molenberghs *et al.*, 2016). Nevertheless, the cerebellum—which contains three times more neurons than the cerebrum—might be more critical to human social cognition than assumed so far. Because it has been traditionally assumed that the cerebellum is involved in motor processing, the contribution of this major part of the brain in social processing has been essentially ignored and therefore remains unexplored.

A recent work has strongly changed that position. In a large-scale meta-analysis on social cognition and the cerebellum that included over 350 functional magnetic resonance imaging (fMRI) studies with healthy humans, Van Overwalle *et al.* (2014) found robust activation of the cerebellum during social judgments, including mentalizing about others’ intentions and beliefs, personality traits and mental time travel in the past and future. Moreover, research revealed that there is a strong neural interaction between the cerebellum and cerebrum during social mentalizing, as revealed by a recent meta-analytic connectivity study on social cognition (Van Overwalle *et al.*, 2015b) as well as a connectivity study pooled across five fMRI studies (Van Overwalle and Mariën, 2016; Van Overwalle *et al.*, 2019). These studies revealed connectivity between the posterior cerebellum and cortical mentalizing areas, including the medial prefrontal cortex (mPFC) and the temporoparietal junction. At a more general level, based on resting-state connectivity for a total sample of 1000 human participants, Buckner *et al.* (2011) identified a distinct default network in the cerebellum that was directly connected to the default network in the cerebrum and that largely overlaps with cerebellar activation during social mentalizing (Van Overwalle *et al.*, 2015a). Part of this mentalizing/default network was located in the posterior cerebellum, just like the above-mentioned task-based connectivity analyses (Van Overwalle *et al.*, 2015b; Van Overwalle and Mariën, 2016).

Although progress has been made in acknowledging the importance of the cerebellum in social cognition, its specific functional role in social mentalizing remains unclear. Via which mechanism does the cerebellum exert its influence on social mentalizing? At a general level, a number of authors have argued that the primary function of the cerebellum is to support sequence learning and memories that underpin skill and motion acquisition, which develop slowly with practice and are inaccessible to consciousness (Ito, 2008; Ferrucci *et al.*, 2013; Leggio and Molinari, 2014; Pisotta and Molinari, 2014). To do this, the cerebellum constructs internal models of motor processes involving sequencing and planning of actions in order to automate and fine-tune voluntary motor processes. These internal models are based on simulations of repetitive patterns of temporally structured events, including motor planning and its sensory consequences. They are used to make predictions about current and future action sequences and continuously send signals to the cerebrum to check whether these anticipations fit with current motion and behavior and its sensorimotor consequences (Leggio and Molinari, 2014). It is assumed that during evolution, a more advanced function developed that allowed the cerebellum to construct internal models of purely mental processes in which event sequences play a role, without overt movements and somatosensory responses (Ito, 2008; Leggio and Molinari, 2014; Pisotta and Molinari, 2014).

With respect to social cognition, the sequencing role of the cerebellum is perhaps most evident and prominent in mental reconstructions of past and future events and in high-level trait inferences based on integrating behavioral events. These social judgments recruit the cerebellum most strongly (see meta-analysis by Van Overwalle *et al.*, 2014). One might argue that in order to make adequate time and trait inferences, it is imperative that sequences of actions can be imagined and integrated into a meaningful impression or judgment. By doing so, the cerebellum allows humans to better anticipate action sequences in an automatic and intuitive way during social contact and interaction, to fine-tune these anticipations and to instantaneously detect disruptions in action sequences. This is essential to understand and predict social behaviors.

Studies with cerebellar patients have begun to address dysfunctions of social cognition in comparison with healthy controls. Some studies provided behavioral stories implying a mental state or belief by the protagonist and reported mixed findings. Sokolovsky *et al.* (2010) reported impairments in some but not all cerebellar patients using a theory-of-mind story task (Blair and Cipolotti, 2000; Van Harskamp *et al.*, 2005), while Roca *et al.* (2013) found no significant differences in mentalizing using the Faux Pas test (Baron-Cohen *et al.*, 1999). In addition, Hoche *et al.* (2016) found worse performance on the Reading the Mind in the Eyes test (Baron-Cohen *et al.*, 2001).

In a first study on action sequencing, Leggio *et al.* (2008) presented cartoon-like drawings and verbal sentences in a random order, and participants had to reproduce them in a plausible behavioral sequence. The authors reported that cerebellar patients performed worse on both sequencing tasks. Using a very similar task with action photos, Cattaneo *et al.*(2012) reported that cerebellar patients performed significantly worse, especially on photos of biological action and less so on physical/mechanical movements. These latter two studies on action sequencing clearly point to the potential diagnostic value of tasks in which an adequate chronological order of actions has to be generated. However, these studies did not enforce participants to infer others’ beliefs, so that it remains unclear to what extent sequence generation during mentalizing rather than simple action observation is the key deficit in cerebellar patients.

False beliefs are a key test for measuring mentalizing. In a typical false-belief story, an object is displaced or changed unbeknownst to the protagonist, so that the participant has to infer a mental belief of the protagonist, which deviates from reality and is thus `false’. False beliefs are a crucial test of the capacity to mentalize because participants must have the understanding that another person may hold mental states or beliefs that are contradicted by reality and different from their own beliefs. To provide a powerful diagnostic test of the sequencing role of the cerebellum, we used a sequential version of a false-belief task in which participants have to generate the correct order of an event, in particular, the picture sequencing task with cartoon-like drawings developed by Langdon and Coltheart (1999) (Figure 1), which was inspired by an earlier version of this task (Baron-Cohen *et al.*, 1986). In contrast to earlier sequencing research that did not distinguish between action observation and mentalizing (Leggio *et al.*, 2008; Cattaneo *et al.*, 2012), this test distinguishes between actions involving physical/mechanical movements, social scripts and false beliefs.

**Fig. 1:**
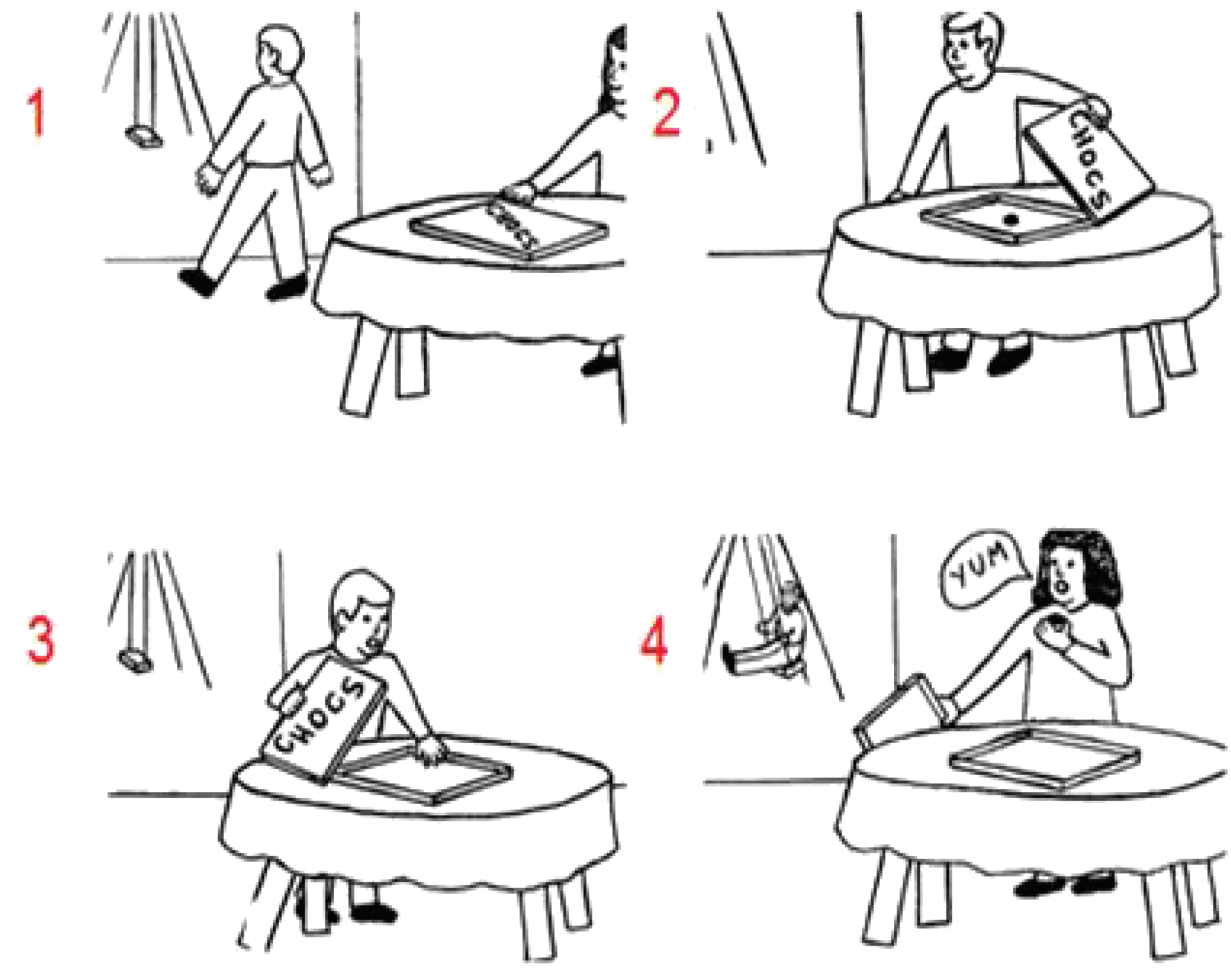
An example of a false-belief sequence in the picture sequencing task (Langdon and Coltheart, 1999; the correct order is 2–1–4–3; the numbers are not shown to the participants but given here for display purposes). Participants had to select, in the correct order, the first picture on the screen, then the second picture and so on. Each time, the pictures moved in the order indicated by the participant.

Another highly important social ability is making trait attributions, that is, inferring the kind of personality trait that someone has by weighting and integrating multiple past behaviors. Making trait attributions is a highly abstract judgment, which may depend in part on a good understanding of action sequencing. For instance, a person may appear less aggressive after learning that prior to his or her aggressive act, the person was strongly provoked or acted in self-defense. Some support for this suggestion comes from a study by Baetens *et al.* (2013) who compared trait inferences against visual descriptions on the basis of a persons’ behaviors presented visually on a photo (e.g. a person reading a book), which allows some room for imaging what went on, and observed strong cerebellar activity.

To investigate the role of sequencing in social cognition, we compared the performance of cerebellar patients and matched healthy volunteers on a whole range of social judgments, some of which required our participants to reconstruct a consistent order of social events (i.e. picture sequencing), to integrate several events into an abstract inference in which sequencing is also important (trait attribution) and to understand a single event that is already ordered (i.e. Dewey Social Stories Test). All patients suffered from a degenerative cerebellar disease, which affected the whole cerebellum. Our hypothesis is that generating appropriate action sequences, and especially false beliefs and abstract traits, depends on the proper functioning of the cerebellum much more than the reconstruction of overlearned social and non-social (mechanical) sequences, or non-sequential behaviors, and thus will reveal the strongest detrimental effects among cerebellar patients.

## Method

### Participants

The final sample consisted of 11 patients with a primary neurodegenerative ataxia or injury to the cerebellum and 9 healthy controls who were volunteers, in many cases the partner of the patient. For all patients, detailed history was elicited and neurological examination and diagnosis performed (by their regular cerebellar medical specialist, Mario Manto). All the patients underwent a brain MRI (see Table 1 for a detailed description of patient diagnosis). The healthy controls were matched for age and other general abilities described below (Table 2). Patients and healthy participants were excluded when they had additional neurologic (in particular Parkinson’s disease or dementia), psychiatric, developmental or severe visual or auditory perception disorders. Patients with essential tremor were included on the basis of recent neuropathological findings demonstrating that this common disorder is associated with a pathology of Purkinje cells (Louis *et al.*, 2016; Kuo *et al.*, 2017). Two additional individuals (one from each group) were removed from the final analysis because they failed these exclusion criteria.

**Table 1:** Diagnosis and brain damage of the patients

Patient	Diagnosis	Brain MRI
1	Sporadic cerebellar ataxia	Diffuse cerebellar cortical atrophy
2	Essential tremor	Normal
3	Fahr syndrome	Calcifications of dentate nuclei and basal ganglia, moderate atrophy of the cerebellar cortex
4	Dominant ataxia (SCA14)	Diffuse cerebellar cortical atrophy
5	Idiopathic late-onset cerebellar ataxia	Diffuse cerebellar cortical atrophy
6	Idiopathic late-onset cerebellar atrophy	Diffuse cerebellar cortical atrophy
7	Essential tremor	Normal
8	Cerebellar ataxia and axonal neuropathy	Diffuse cerebellar cortical atrophy
9	Cerebellar ataxia (paraneoplastic syndrome)	Diffuse cerebellar cortical atrophy
10	Autosomal recessive spastic ataxia of Charlevoix–Saguenay	Diffuse cerebellar cortical atrophy
11	Spinocerebellar ataxia 8	Slight atrophy of the cerebellar cortex

Note: Patient 3 was included in part based on recent evidence that the basal ganglia and cerebellum are anatomically connected and form the same network (Bostan and Strick, 2018)

**Table 2:** Characteristics of patients and controls (means and tests)

	Mean		*t*-test		M–W test	Effect size
Variables	Patients (*n* = 11)	Controls (*n* = 9)	*t*-value	*P*-value	*P*-value	Hedges *g*
Age (in years)	62.55	64.00	0.220	0.414	0.904	0.102
Gender (% female)	45%	67%	0.921	0.185	0.456	0.414
MMSE (total score)	28.27	29.00	0.916	0.186	0.230	0.412
CCAS (total score)	84.20	92.50	1.110	0.142	0.274	0.526
Category Switching (% failure)	50%	13%	1.721	0.052°	0.203	0.816
Digit Span forward (% failure)	50%	0%	2.667	0.008**	0.083°	1.265
BDI-II (total score)	10.45	6.88	1.081	0.147	0.351	0.502

Note: `% failure’ refers to % below the cut-off point recommended by Hoche *et al.* (2018)

°*P* ≤ 0.10, *P ≤ 0.05, **P ≤ 0.01 given a one-sided *t*-test/M–W U test

All patients and controls were French-speaking except for one Dutch-speaking individual in each group. Therefore, all tests, which were developed and available in Dutch, were translated into French, which was checked by backtranslation into Dutch (in both directions by native professional translators), unless noted otherwise (when an existing French version was already available).

This study was approved by the Ethics Committee of the Université Libre de Bruxelles and Vrije Universiteit Brussel (Belgium), and an informed consent was obtained from all participants.

### Cognitive and motor tests

All cognitive and motor (i.e. ataxia) tasks were presented on paper, and existing Dutch and French versions were used, unless noted otherwise. Given the motor impairments of the participants, a research assistant read the questions and stimulus material out loud and noted the answers given by the participant. To check whether healthy controls and patients were matched with respect to general functioning, we took the following tests:

- Mini-Mental State Examination (MMSE; Folstein *et al.*, 1975). The MMSE measures several executive cognitive functions including time and space orientation, short and long term memory, attention, language, computing, practice and visual construction. The MMSE can detect subtle cognitive impairment and research showed average to high reliability and high validity (Tombaugh and McIntyre, 1992).
- Cerebellar Cognitive Affective Syndrome Scale (CCAS). This recently developed short (10–12 min) neuropsychological test (Hoche *et al.*, 2018) can identify cognitive and affective impairments of cerebellar patients, including deficits in executive function, language, visual spatial function and neuropsychiatric features including impairments in attentional control, emotional control, psychosis spectrum disorders and social skills. This test was translated from the original English version into Dutch and French, with backtranslation to English.
- Beck Depression Inventory Second Edition (BDI-II) was administered to test for depression. This is a self-report 21-item questionnaire, which measures the presence of depressive symptoms and exhibits high test–retest reliability (*r* = 0.93) and high internal consistency (Cronbach alpha, 0.92–0.93; Beck *et al.*, 1996).
- Scale for the Assessment and Rating of Ataxia (SARA; Schmitz-Hubsch *et al.*, 2006) is a clinical scale, which assesses a range of different motor impairments in cerebellar ataxia. The scale is made up of eight items related to gait, stance, sitting, speech, finger–chase test, nose–finger test, fast alternating movements and heel–shin test.

As shown in Table 2, a parametric *t*-test and non-parametric Mann–Whitney (M–W) test revealed no significant differences between the patient and control groups on the cognitive tests described above, as well as on other demographic variables. The effects size of the differences measured by Hedges *g* was small to medium (all <0.53). However, two executive subtests of the novel CCAS revealed (close to) significant impairments of cerebellar patients compared to healthy controls when using the pass-fail cut-offs recommended by Hoche *et al.* (2018). This included the subtests Category Switching (*P* = 0.052) and Digit Span (*P* < 0.01; Table 2).

### Tests of social understanding

We selected a number of social cognitive tasks that reliably elicit cerebellar activation (Van Overwalle *et al.*, 2014), some of which require understanding and generation of chronological sequences. Unless noted otherwise, these tasks were programmed in Eprime 2.0 and presented on a Window Surface tablet that was controlled by touching the appropriate response; the tablet was connected to a larger screen to facilitate viewing the material. Given the severe motoric difficulties of the participants, a research assistant read the questions and stimulus material out loud and controlled the tablet by touching the response given by the participant. All tasks were developed or available in Dutch and translated to French with backtranslation to Dutch. Given the severe impairments of the patient population, most tests require participants to make a choice from available options rather than to generate their own responses.

- Picture sequencing task (Langdon and Coltheart, 1999). Participants watched 12 cartoon-like scenarios that represented 4 mechanical, 4 social script and 4 false-belief events (for an example, Figure 1). The experiment began with two practice trials. Each trial started with a fixation cross (1 s), followed by the presentation of 4 pictures in a random order, and participants had to line the pictures up in the correct order like in a comic strip. They had to indicate the correct order by first selecting the first picture on the screen, then the second picture and so on. Each time, the pictures moved on the screen along the order indicated. At the end of each trial, participants could cancel and redo the trial or end the trial. After each block of 11 trials, participants received a 30 s break that they could end earlier.
- Trait attribution. We borrowed the trait inference task from an earlier study with patients with vmPFC lesions (Kestemont *et al.*, 2016), from which the original trait-implying sentences were kept, but novel forced-choice response options were developed to make the task manageable for our cerebellar patients (Appendix A). Participants read 18 pairs of 2 sentences, each describing a behavior that implied the same trait of a person performing the behavior. Half of the sentences had a positive valence, while the other half had a negative valence. `Star Trek-like’ names were used to avoid similarities with familiar others known to the participants, which may bias the attribution process. The experiment started with two practice trials. Each trial consists of a fixation cross (1 s), and 2 trait-implying sentences followed by a question (`This person is…’). Participants responded by selecting one of four possible personality traits presented on the screen. After each block of 11 trials, participants receive a 30 s break that they could end earlier.The response options involved the correct trait ([Bibr ref23][Bibr ref23], Appendix A2) and three distractor traits of which two were of equal valence and one of the opposite valence, selected from the trait responses on other trait-implying sentences (as pilot tested by Kestemont *et al.*, 2016). We conducted additional pilot tests with healthy university students (*n* = 29) who indicated on a 7 point-scale to what extent these traits were not applicable (= 0) to applicable (= 7). We selected only sentences for which the correct traits were judged to be quite applicable (>5.3) and the distractors less applicable (<4.2), resulting in moderate differences in ratings for the correct trait and the strongest distractor (difference ranging between 1.9 and 4.5, with mean, 3.3). The correct traits were highly reliable (Cronbach’s alpha, 0.88).
- Causal attribution: This task served as control for the trait attribution task because it requires less abstraction than traits and refers only to a current event. We borrowed the causal attribution task from Kestemont *et al.* (2016) mentioned earlier, from which the original cause-implying sentences were kept, but novel forced-choice response options were developed to make the task manageable for our cerebellar patients (Appendix B). Participants read 20 sentences describing several everyday events. As described by Kestemont *et al.* (2016, Appendix A1), half of the sentences involved an event in which the situation was implied as the cause (e.g. `Maldron earns a salary’—implies that this person has a job), while the other half implied the person as the cause (e.g. `Dilla can work well together’—implying that Dilla is social). Orthogonal to this, half of the sentences described a positive event, while the other half described a negative event. Again, `Star Trek-like’ names were used. The experiment started with two practice trials. Each trial consists of a fixation cross (1 s) followed by the event description and a question (`The cause of this event is…’). Participants respond by selecting one of four potential causal explanations offered on the screen. After each block of 11 trials, participants receive a 30 s break that they could end earlier. The response options involved the correct causal response (Kestemont *et al.*, 2016, Appendix A1) and three distractor causes of which one came from the opposite (person *vs* situation) causal category and two came from the same and opposite causal category but with the opposite valence. The distractors were selected in a similar manner as for the trait attributions, that is, from the correct responses on other events (Kestemont *et al.*, 2016, Appendix A1). We conducted a pilot test with healthy university students (*n* = 66) who indicated on a 7 point-scale to what extent these causes were not applicable (= 1) to applicable (= 7). We selected only sentences for which the correct causes were judged very applicable (>6) and the distractors less applicable (<4.5), with at most only one distractor being very implausible (<1.5), resulting in moderate differences in ratings between the correct trait and the strongest distractor (difference ranging between 2.1 and 4.8, with mean, 4.0). An additional pilot test with healthy university students (*n* = 21) indicated that the correct causes were highly reliable (Cronbach’s alpha, 0.83).
- Dewey Social Stories Test (Dewey, 1991). We used the Dutch version of the social stories test (Smit, 2011) and on paper. Participants read eight short stories in which a protagonist engaged in various actions, and the stories were subdivided in a varying number of segments (2–6). Each segment of a story was followed by several questions. First, participants were asked to rate how they thought most people would judge the behavior if they witnessed it, and they answered on a four-response scale to what extent the action by the protagonist in that situation was considered deviant using the scale anchors normal, strange, very strange or shocking. Second, participants were asked an open question about why they thought most people would perceive the protagonist’s behavior in that way. The participants’ open-ended answers were coded according to the implicit coding system developed by Callenmark *et al.* (2014). The first implicit coding, spontaneous perspective taking, reflects the ability to explain behavior using other people’s mental states such as thought, feeling and desire, without being explicitly prompted to. The second implicit coding, implicit social awareness, refers to the internalization of social rules and norms that create a cognitive short-cut when predicting a person’s behavior in a given situation. Agreement between two raters on these implicit codings was substantial in the present study (agreement, 78%; *r* = 0.56)

## Results

All analyses are based on the raw test scores without rescaling or normalization, since we were mainly interested in differences between cerebellar patients and healthy controls. We tested these differences with a parametric *t*-test as well as with a non-parametric M–W test because given the limited number of participants, this test does not require that all parametric conditions are satisfied (e.g. normal distribution). The significance (*P*-value) of the *t*-test was computed one sided because we hypothesized that cerebellar patient would perform worse (not better) than healthy controls on cognitive and social tests (Table 3). We also report the effect size of the group differences using Hedges *g*, the power of the *t*-test as calculated by G*Power (Faul *et al.*, 2007) and a bootstrapping analysis by randomly subsampling from the two groups and presenting the overall *t*-test results. These analyses provide additional evidence on the robustness of signficant differences given the small sample size. Finally, we also conducted all calculations without the Dutch participants, but none of the results were altered appreciably, except when noted otherwise.

**Table 3:** Performance (% correct or score) on social tasks

	Mean		*t*-test			M–W test	Effect size	
Task	Patients	Controls	*t*-value	*P*-value	Bootstrap	*P*-value	Hedges *g*	Power
Sequencing (total score, *n* = 8 & 8)	73%	90%	0.026	0.010**	0.018*	0.015*	1.314	0.52
Mechanical	79%	94%	1.713	0.054°	0.071°	0.234	0.857	0.51
Social script	90%	95%	0.988	0.170	---	0.574	0.494	0.53
False belief	48%	83%	3.432	0.002**	0.004**	0.005**	1.716	0.52
Trait attribution ^a^ (*n* = 11 & 7)	91%	98%	1.403	0.090°	0.080°	0.151	0.678	0.57
Causal attribution (*n* = 11 & 8)	93%	91%	0.766	0.227	---	0.442	0.356	0.79
Dewey Story Test (deviance rating)	9.73	10.56	0.377	0.355	---	1.000	0.169	0.72
Spontaneous perspective	5.05	5.00	0.057	0.478	---	0.656	0.026	0.96
Implicit social awareness	19.82	20.50	0.672	0.255	---	0.766	0.302	0.60

Note: ^a^ omitting outlier with >2 s.d. from group mean of controls. Some tasks were not completed by all participants, as indicated by a reduced number of participants (*n*) for patients & controls, respectively.

°*P* ≤ 0.10, *P ≤ 0.05, **P ≤ 0.01 given a one-sided *t*-test/M–W U test

**Fig. 2:**
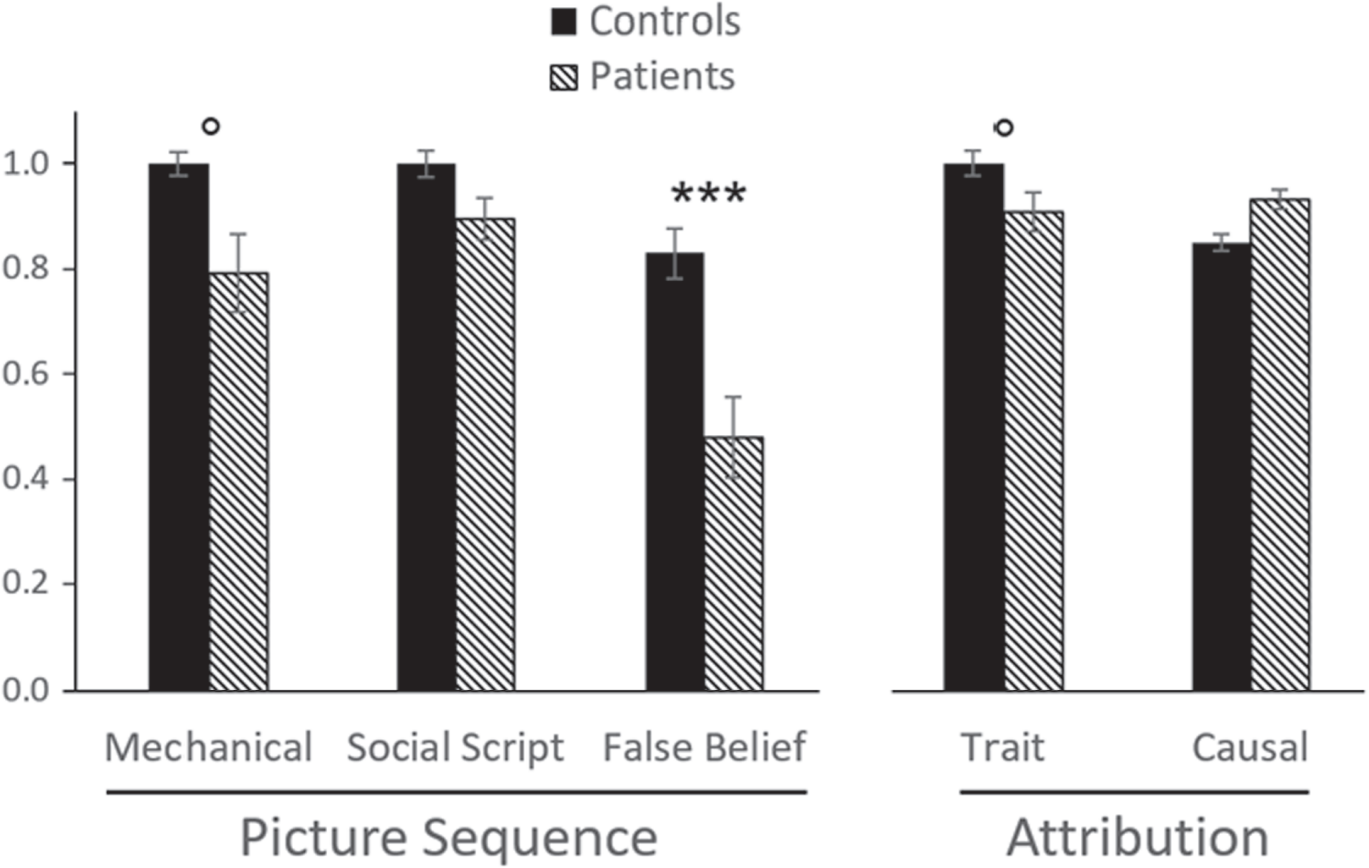
Comparison of degenerative cerebellar patients in comparison with healthy controls on the picture sequencing and attribution tasks. Error bars reflect standard errors. °*P* < 0.10, ***P < 0.002 (one-sided *t*-test). Numbers of participants are reported in Table 3.

The most important results with respect to the hypothesized diagnostic value of tasks with a sequencing and integrative component (i.e. the picture sequencing and trait attribution task) are depicted in Figure 2. As hypothesized, the picture sequencing task reveals significant impairments in cerebellar patients compared to healthy controls, especially for the generation of a correct chronological order of social events that involve false-belief reasoning (*P* < 0.005) and not for overlearned social scripts. Mechanical order was also marginally significant given the more powerful *t*-test (*P* = 0.054), which is to be expected given that the generalized cerebellar degenerative lesions in our patients also impact on non-social sequencing functions. The effect size using Hedges *g* for false beliefs exceeds 1.7 and can be considered large. The power for the picture sequencing subtests was modest (0.51–0.53). A bootstrapping analysis with 5000 resamples confirmed the significant impairment on false-belief sequencing for patients (*P* = 0.004) and the marginally significant difference for mechanical sequencing (*P* = 0.071).

However, contrary to our hypothesis, trait attributions showed only a marginally significant impairment in cerebellar patients compared to healthy controls (*P* < 0.09), after omitting an outlier in the control group with more than 2 standard deviations from the group mean, while causal attributions that served as a control task did not show any effect, as predicted. The effect size using Hedges *g* was medium for trait attributions and weak for causal attributions, while power was modest (0.57) and at a conventional level (0.79), respectively. A bootstrapping analysis with 5000 resamples confirmed that there was a marginally significant difference for trait attributions (*P* = 0.080) Computations without the Dutch participants revealed that the marginal effect of the trait attribution task now fell below significance (*P*_*t*-test_ = 0.121).

The other social task involving the understanding of social events (Social Story Test; Dewey, 1991), but without a reconstructive sequencing or integrative component, revealed no significant differences between cerebellar patients and healthy controls nor a trend in the hypothesized direction. The effect sizes are at or below 0.30, and power ranged from modest to high (0.60–0.96).

For exploratory purposes, we also computed a Pearson correlation between the degree of motor ataxia (SARA, *n* = 11) and performance on the cognitive and social tests. As one might expect, the results showed (near) significant correlations with cognitive tests including the MMSE (*r* = −0.63, *P* < 0.05) and CCAS (*r* = −0.60, *P* = 0.066), but also showed significant correlations with social tests including mechanical sequencing (*r* = −0.75, *P* < 0.05), causal attribution (*r* = −0.67, *P* < 0.05) and the Social Story Test (deviance score: *r* = 0.75, *P* < 0.01; implicit social awareness: *r* = −0.83, *P* < 0.005). Given that we had no a priori hypotheses and significance levels are not corrected for multiple comparisons, these results should be treated with caution.

## Discussion

This study investigated whether social mentalizing, a form of higher-order social cognition unique to humans, is impaired in cerebellar patients (cf. Van Overwalle *et al.*, 2014) and to what extent the cerebellar function of learning and automatizing action sequences (e.g. Leggio and Molinari, 2014) is crucial in this process. In particular with respect to social mentalizing, we tested the hypothesis that the cerebellum is involved in learning and automatizing the understanding of social action sequences that are indicative or dependent of a person’s mental state. To test this hypothesis, we investigated not only the sequential reconstruction of physical or social movements or actions as was done in earlier research (e.g. Leggio *et al.*, 2008; [Bibr ref12][Bibr ref12]), but also of social actions in terms of the mental state of the protagonist (e.g. involving beliefs or abstract traits). This hypothesis predicts that the reconstruction of a correct chronological order of social events involving mentalizing (false-belief reasoning) would be most impaired in cerebellar patients and less so for overlearned social scripts or for mechanical event order. Moreover, trait attributions inferred from behavioral descriptions were also predicted to reveal impairments in cerebellar patients on the suggestion that trait inferences require to integrate several behaviors into an abstract social inference that also requires to take sequencing into account (e.g. a person acting out in self-defense rather than an impulse will be judged to be relatively unaggressive). In contrast, we expected little impairments in cerebellar patients in test of social mentalizing when this active reconstruction or integration of sequencing was absent.

The results strongly supported our hypothesis that the reconstruction of action sequences in their chronological order, as revealed by the results on the picture sequencing task (Langdon and Coltheart, 1999), was most impaired among our cerebellar patients for false-belief stories, while mechanical or overlearned social scripts were little or not affected. The sequencing task showed significant impairments in cerebellar patients for false-belief stories that is consistent with our hypothesis that the posterior cerebellum is responsible for learning and automatizing social action sequences that involve the protagonist’s mental state. Trait attributions (Kestemont *et al.*, 2016) showed only a marginal impairment with the more powerful parametric test, but not with the non-parametric test. Because the statistical power for the trait attribution and picture sequencing tests was equally modest, power is not a likely explanation for the weak trait attribution results.

On the other hand, as hypothesized, the other mentalizing tasks that lacked a clear active sequencing component (in which participants have to generate a correct action sequence) did not reveal robust differences between cerebellar patients and healthy controls, including theory-of-mind story understanding (Dewey, 1991), and causal attributions of here and now events (Kestemont *et al.*, 2016). These results demonstrate that although these remaining social tasks require elements of mentalizing, they all lack a clear sequencing component so that they do not elicit or require the working of the cerebellum. This is consistent with the weak and conflicting findings from earlier research with cerebellar patients using theory-of-mind story tasks without sequential component (Sokolovsky *et al.*, 2010; Roca *et al.*, 2013; Hoche *et al.*, 2016). This interpretation is also supported by a recent fMRI study involving the picture sequencing test, which confirmed the strong involvement of the posterior cerebellum in belief sequences as opposed to mechanical and social script routines (Heleven *et al.*, 2018).

The marginal finding for trait attributions is promising and consistent with our suggestion that trait attributions might reflect a persons’ permanent internal state involving high-level abstract summaries of actual behaviors for which sequencing might also be important. However, this test provided a sequence of behavioral descriptions, but did not require an active sequencing process to generate a correct order, which may explain why its effect is not so robust. This explanation is consistent with the meta-analysis by Van Overwalle *et al.* (2014), where cerebellar activation was often found in person trait studies involving video clips and stories (i.e. sequences), but only in one-third of the person trait studies overall. Why many studies failed to show significant cerebellar activation even when they contained behavioral actions like in our study is still unclear.

In the picture sequencing test, we observed that mechanical sequences (e.g. a truck hitting a stone, which roles down the hill and then hits a tree) show almost significant differences between groups, while there are no differences in the social scripts sequences (e.g. going to the groceries, picking some items and pay). Given that our patients have generalized cerebellar impairments, we expected some weaker performance in these two non-mentalizing conditions because they both reflect well-learned routines, which require minimal deployment of the cerebellum. Perhaps the limited number of participants in this pilot study, as well as potential similarities between the mechanical and social script conditions (e.g. a social script of brushing teeth is also a physical action), may additionally explain why these conditions show quite similar non-significant results. However, these additional explanations are unlikely because the fMRI study mentioned earlier (Heleven *et al.*, 2018) involved many more participants and stimulus materials but showed no significant differences between mechanical and social script conditions.

Although we had expected to find some minimal impairments among cerebellar patients on the other mentalizing tasks including causal attribution (Kestemont *et al.*, 2016) and theory-of-mind story understanding (Dewey, 1991), none were found. This might be due to the limited number of participants, so that some dysfunctions could have been identified with more data included from patient and control groups. Nevertheless, it is interesting to note that within patients, level of motor impairment (i.e. ataxia) was significantly related to these two social tests as well as with mechanical sequencing. These results seem to suggest that cerebellar motor impairment might be related to social deficits. Although this might seem surprising because one might expect such result mainly for social judgments based on mirroring (i.e. sensorimotor network involved in sequence learning) but not for beliefs inference (i.e. mentalizing network involved in causal attribution and theory of mind), it can be explained by the fact that these were patients with widespread cerebellar deficits, with impairment in (anterior) motor as well as in (posterior) social cerebellar areas. This interesting result is, however, provisional because it is limited to the patient population, does not involve corrections for multiple testing and is merely exploratory.

Because the statistical effects of the picture sequencing test were so robust and compelling in the comparison between cerebellar patients and healthy controls, we decided to continue our research by exploring this specific sequencing finding further, rather than continuing the whole set of present tests. This seemed the most rational and productive choice, one which is also consistent with an ethical concern not to overburden our patients with tests that have little diagnostic value. Obviously, more research is needed to determine whether our sequencing hypothesis and the current picture sequencing test are sufficiently robust and diagnostic for identifying social cerebellar dysfunctions, and what their potential limitations and shortcomings are.

There are indeed limitations in the current work. First, it is still unclear whether the false-belief stories induced strong impairments among cerebellar patients because these stories involve false beliefs or because they are relatively novel (regardless of whether true or false beliefs were involved). A particular aspect of the false-belief stories in the picture sequencing task is that they are surprising and unfamiliar to the participants, while the social scripts reflect well-known action sequences (e.g. brushing your teeth and shopping at the groceries). To answer this critical limitation, it is necessary to extend the picture sequencing task (Langdon and Coltheart, 1999) with a true-belief condition matched for novelty with the false-belief condition.

A second limitation is that the sequencing hypothesis was only robustly tested and demonstrated given cartoon-like pictures. Would the sequencing hypothesis uphold when using verbal versions of the sequencing task as well? This is a critical question for the sequencing hypothesis, although prior findings from Leggio *et al.* (2008) suggest that verbal sequencing material also reveals cerebellar dysfunctions. These two limitations were addressed in the fMRI study mentioned earlier (Heleven *et al.*, 2018). The results showed little differences in brain activation between true and false beliefs and very similar results in comparison with mechanical and social script routines across the picture sequencing test and a verbal version of it. However, these results need further confirmation by patient studies. A related limitation is that the trait attribution task had very limited diagnostic value for identifying social cerebellar dysfunction. Perhaps this task needs stronger sequencing components in order to become more diagnostic, for instance, by making it more predictive or reconstructive like the picture sequence task.

A third limitation admitted earlier is that the sample contained patients with widespread cerebellar damage. This widespread defect potentially affected both motor and non-motor functions and makes it impossible to draw firm conclusions on the source of the observed social sequential impairments. Nevertheless, the fMRI study mentioned earlier (Heleven *et al.*, 2018) using the same sequencing test confirmed that social belief sequencing recruits the posterior cerebellum. Another, related limitation acknowledged before, is the limited sample size. Our analysis suggested that this reduced the power of our statistical *t*-tests and so may have reduced potential observed differences below a conventional significance threshold. We therefore applied resampling bootstrapping to gain more information on how robust the significant *t*-test differences really were, and this analysis confirmed that false-belief sequencing showed significant decrements in performance of our patients compared to healthy controls, as well as the other marginal differences. Another way to overcome this shortcoming is by using one-to-one matching between patients and controls, which would ensure higher comparability between patients and controls and would reduce variability and systematic differences due to background variables that are not of interest (Everitt and Palmer, 2005). However, this matching strategy is difficult to achieve for the present study given that spouses of the patients most often served as controls and thus introduce gender as confound. Perhaps this is a possibility for future research. Another way to address the limited sample size is to collect data from a larger control group, so that comparisons between patients to a `normal distribution’ would be possible. As noted above, this is the approach we are currently pursuing, together with some additional patients, on the most promising social tests.

A final limitation is that the Schmahmann scale (CCSA), which was specifically developed to diagnose cerebellar patients, did not show significant differences between our patient and healthy groups, except for two subtests involving executive control (i.e. Category Switching and Digit Span). Consequently, it is possible that our patients had atypical cerebellar dysfunctions, which may perhaps explain why they were strongly impaired on the sequencing test. If so, this may potentially limit the generalizability of the present findings but raises important question with respect to cerebellar subgroups who may suffer from elevated sequencing dysfunctions.

The present research on cerebellar dysfunctions has widespread clinical implications. The cerebellum has been found to be crucially implicated in the pathophysiological mechanisms subserving a broad range of neuropsychiatric and neurodevelopmental disorders such as autistic spectrum disorders, attentional deficit and hyperkinetic disorder, depression and schizophrenia (Bauman and Kemper, 2005; Penn, 2006; Wang *et al.*, 2014; D’Mello *et al.*, 2015). Identification of the role of the cerebellum in social cognition may open very promising avenues for future clinical diagnostics and treatment, with diagnosis including social mentalizing tasks and treatments that target this specific deficit. Up till now, social tests do not constitute an inherent part of the diagnostic work-up and treatment of cerebellar impairments, and patients may receive inadequate support because of this neglect.

## Supplementary Material

scan-18-186-File001_nsz032

scan-18-186-File002_nsz032

## Appendix A

Trait attributions (best possible English translation from Dutch)

**Table:**

Valence and sentences		Correct	Distractors		
Negative		CorrectTrait	DifferentTrait	Different Trait	Different Valence
Hanstra prefers to do many things alone	never goes out of her house	asocial	unfair	unreliable	generous
Quilot never gives her opinion	rarely formulates her thoughts	introverted	unfriendly	melancholic	helpful
(Ybar rarely helps an old man)	doesn’t repair his mother’s broken chair	unhelpful	melancholic	aggressive	reliable
Kerbol did not return his sister’s doll	gave his father a mock report	unreliable	aggressive	asocial	honest
Terp gave his mother the wrong test	didn’t give his grandmother her money	unreliable	aggressive	asocial	honest
Niav asked his team to cheat	asked his teammate to take dope	unfair	asocial	unfriendly	responsible
Melsu insulted her colleague	made a bad remark to her colleague	unfriendly	extroverted	irresponsible	funny
Bloemak is crying a lot	is seldom laughing	melancholic	unhelpful	stingy	sweet
Kavlim is often very sad	doesn’t knows any jokes	melancholic	unhelpful	stingy	sweet
Positive					
Blublo calculated the tax very fair	calculated the income very precisely	reliable	sweet	social	unfair
Gimar calculated the game very correctly	calculated the equipment very appropriately	reliable	sweet	social	unfair
Bollap talks to people on the train	tells about her thoughts	extroverted	responsible	helpful	melancholic
Brimasy tells a lot when in a pub	talks about the nice vacation	extroverted	responsible	helpful	melancholic
Elkmo tells good jokes	grapples his friend	funny	generous	reliable	aggressive
Vousblo plays some nice sketches	is often laughing	funny	generous	reliable	aggressive
Burc showed her admiration to the speaker	smiled at the woman	sweet	reliable	honest	asocial
Wemblo follows his schedule perfectly	acts with proper approach	responsible	helpful	friendly	unfriendly
Ismin gave millions to 11-11-11	gave his golden watch to the concierge	generous	extroverted	funny	unhelpful

Note: The name of the protagonist is repeated in the second sentence but now shown here.

## Appendix B

Causal attributions (best possible English translation from Dutch)

**Table:**

Conditions, valence and sentences	Correct Response	Distractors		
Person -	Person -	Situation -	Person +	Situation +
Radro plays with her feelings	playboy	wet	optimistic	sports
Xapo goes to the hospital	ill	smelly	social	Easter
Neesuw never talks to someone	asocial	much noise	happy	has leave
Kobil calls emergency service for minor injuries	anxious	puncture	honest	holiday
Telwor sleeps on the sofa	tired	broken	warm	work
Person +	Person +	Situation +	Person −	Situation −
Fafel doesn’t lose his courage	persistent	dinner time	pedophile	sour milk
Knarf talks to his colleagues	social	party	anger	breakdown
Loma thinks about his girlfriend	in love	with family	psychopathic	accident
Tarin thinks that the future is beautiful	optimistic	sports	playboy	wet
Mart carries the luggage of the children	helpful	traffic rules	tired	water is cold
Situation -	Situation −	Person −	Situation +	Person +
Birmak pushes the car	breakdown	anger	party	social
Cyralis pushes the motorbike	breakdown	psychopathic	nice singing	sweet
Ashram shives from the wind	it's cold	aggressive	at destination	helpful
Pheldar pulls the brake	danger	tired	work	warm
Stelvine replaces the tire	flat tire	chatting	birthday	social
Situation +	Situation +	Person +	Situation −	Person −
Eelram listens to the singing	nice singing	sweet	breakdown	aggressive
Xoyrish undresses in the locker room	sports	optimistic	wet	playboy
Listek can go on a holiday	has leave	social	much noise	asocial
Alnorak swims in the Mediterranean	holiday	helpful	broken	anxious
Maldron earns a salary	work	social	danger	tired

Note: + means positive; −, negative.
