# Supplementary material for: The role of the cerebellum in reconstructing social action sequences: a pilot study
Source: Soc Cogn Affect Neurosci. 2019 Apr 30;14(5):549–58. doi: 10.1093/scan/nsz032 (PMC6545532; doi:10.1093/scan/nsz032)
Supplement: scan-18-186-File001_nsz032 [file scan-18-186-file001_nsz032.doc]

|  | | |
| --- | --- | --- |
|  |  |  |
|  |  |  |
|  |  |  |
|  |  |  |
|  |  |  |
|  |  |  |
|  |  |  |
|  |  |  |
|  |  |  |
|  |  |  |
|  |  |  |
|  |  |  |
|  | | |

|  | | | | | | |
| --- | --- | --- | --- | --- | --- | --- |
|  |  | |  | |  |  |
|  |  |  |  |  |  |  |
|  |  |  |  |  |  |  |
|  |  |  |  |  |  |  |
|  |  |  |  |  |  |  |
|  |  |  |  |  |  |  |
|  |  |  |  |  |  |  |
|  |  |  |  |  |  |  |
|  |  |  |  |  |  |  |
|  | | | | | | |

|  |  | | | | | | | | |
| --- | --- | --- | --- | --- | --- | --- | --- | --- | --- |
|  | |  | |  | | |  |  |  |
|  | |  |  |  |  |  |  |  |  |
|  | |  |  |  |  |  |  |  |  |
|  | |  |  |  |  |  |  |  |  |
|  | |  |  |  |  |  |  |  |  |
|  | |  |  |  |  |  |  |  |  |
|  | |  |  |  |  |  |  |  |  |
|  | |  |  |  |  |  |  |  |  |
|  | |  |  |  |  |  |  |  |  |
|  | |  |  |  |  |  |  |  |  |
|  | |  |  |  |  |  |  |  |  |
|  |  | | | | | | | | |
|  |  | | | | | | | | |

: Trait Attributions (best possible English translation from Dutch)

|  | |  |  | | | |
| --- | --- | --- | --- | --- | --- | --- |
|  |  |  | |  |  |  |
|  |  |  | |  |  |  |
|  |  |  | |  |  |  |
|  |  |  | |  |  |  |
|  |  |  | |  |  |  |
|  |  |  | |  |  |  |
|  |  |  | |  |  |  |
|  |  |  | |  |  |  |
|  |  |  | |  |  |  |
|  |  |  | |  |  |  |
|  |  |  | |  |  |  |
|  |  |  | |  |  |  |
|  |  |  | |  |  |  |
|  |  |  | |  |  |  |
|  |  |  | |  |  |  |
|  |  |  | |  |  |  |
|  |  |  | |  |  |  |
|  |  |  | |  |  |  |
|  |  |  | |  |  |  |
|  |  |  | |  |  |  |

**Supplementary Material:** Scores for each cognitive and social test

|  |  |  | | |
| --- | --- | --- | --- | --- |
|  |  |  |  |  |
|  |  |  |  |  |
|  |  |  |  |  |
|  |  |  |  |  |
|  |  |  |  |  |
|  |  |  |  |  |
|  |  |  |  |  |
|  |  |  |  |  |
|  |  |  |  |  |
|  |  |  |  |  |
|  |  |  |  |  |
|  |  |  |  |  |
|  |  |  |  |  |
|  |  |  |  |  |
|  |  |  |  |  |
|  |  |  |  |  |
|  |  |  |  |  |
|  |  |  |  |  |
|  |  |  |  |  |
|  |  |  |  |  |
|  |  |  |  |  |
|  |  |  |  |  |
|  |  |  |  |  |
|  |  |  |  |  |

| Patiënt | MMSE | CCAS | CCAS CatSwitch | CCAS fDigSpan | BDI | Sequence all % | Sequence mechanical % | Sequence social script % | Sequence belief % | Trait % | Causal % | DST_SS | DST_ISA | DST_SPT | SARA |
| --- | --- | --- | --- | --- | --- | --- | --- | --- | --- | --- | --- | --- | --- | --- | --- |
| 1 | 27 |  | 8 | 5 | 3 | 0.41 | 0.33 | 0.67 | 0.13 | 0.67 | 0.8 | 14 | 14.5 | 6 | 44 |
| 2 | 29 | 87 | 15 | 5 | 25 | 0.89 | 1 | 1 | 0.67 | 0.94 | 0.9 | 9 | 19 | 2 | 16 |
| 3 | 26 | 81 | 12 | 5 | 18 |  |  |  |  | 0.94 | 1 | 4 | 20.5 | 4.5 | 14 |
| 4 | 29 | 97 | 15 | 5 | 18 | 0.93 | 0.88 | 1 | 0.92 | 1 | 0.9 | 8 | 18.5 | 8 | 27 |
| 5 | 30 | 93 | 6 | 6 | 1 | 0.71 | 0.88 | 0.75 | 0.5 | 0.94 | 0.9 | 10 | 22 | 5 | 9 |
| 6 | 30 | 98 | 13 | 8 | 7 | 0.85 | 1 | 1 | 0.54 | 1 | 0.95 | 8 | 21.5 | 6 | 21 |
| 7 | 29 | 92 | 4 | 6 | 5 | 0.74 | 1 | 1 | 0.21 | 0.89 | 1 | 5 | 21.5 | 5.5 | 43 |
| 8 | 29 | 53 | 7 | 6 | 4 | 0.67 | 0.58 | 0.88 | 0.54 | 0.67 | 0.95 | 10 | 21.5 | 6 | 14 |
| 9 | 24 | 52 | 9 | 7 | 7 | 0.63 | 0.67 | 0.88 | 0.33 | 1 | 0.9 | 18 | 14.5 | 6.5 | 13 |
| 10 | 29 | 96 |  |  | 10 |  |  |  |  | 1 | 0.95 | 9 | 22 | 1.5 | 19 |
| 11 | 29 | 93 | 12 | 5 | 17 |  |  |  |  | 0.94 | 1 | 12 | 22.5 | 4.5 | 18 |
| Control |  |  |  |  |  |  |  |  |  |  |  |  |  |  |  |
| 1 | 30 | 92 | 11 | 6 | 12 | 0.75 | 0.83 | 1 | 0.67 | 0.94 | 0.85 | 7 | 20.5 | 2.5 |  |
| 2 | 30 | 99 | 15 | 6 | 2 | 0.95 | 1 | 0.83 | 1 | 1 | 0.95 | 12 | 21.5 | 4 |  |
| 3 | 30 | 92 | 13 | 7 | 6 | 0.96 | 1 | 1 | 0.88 | 1 | 0.9 | 23 | 20 | 8 |  |
| 4 | 28 | 82 | 10 | 7 | 6 | 0.81 | 0.92 | 0.875 | 0.625 | 0.94 | 0.95 | 5 | 19.5 | 6.5 |  |
| 5 | 29 | 66 | 7 | 6 | 4 | 0.88 | 0.88 | 1 | 0.75 | 0.79 | 0.95 | 9 | 21 | 4.5 |  |
| 6 | 25 |  |  |  |  |  |  |  |  |  |  | 16 | 20.5 | 4 |  |
| 7 | 30 | 106 | 15 | 6 | 19 | 0.9 | 0.92 | 0.875 | 0.92 | 0.94 | 0.95 | 7 | 20.5 | 5.5 |  |
| 8 | 30 | 106 | 15 | 7 | 6 | 1 | 1 | 1 | 1 | 1 | 0.9 | 11 | 22 | 5 |  |
| 9 | 29 | 97 | 15 | 6 | 0 | 0.96 | 1 | 1 | 0.83 | 1 | 0.85 | 5 | 19 | 5 |  |
| Note: % = percentage correct; MMSE = Mini Mental State Examination; CCAS = Cerebellar Cognitive Affective Syndrome Scale; CatSwitch = Category Switching (% failure); fDigSpan = Forward Digital Span (% failure); BDI = Beck Depression Inventory; DST = Dewey Social Stories Test; SS = social deviance score; ISA = Implicit Social Awareness; SPT = Spontaneous Perspective Taking, SARA = Scale for the Assessment and Rating of Ataxia. | | | | | | | | | | | | | | | |
