# Supplementary material for: The role of the cerebellum in reconstructing social action sequences: a pilot study
Source: Soc Cogn Affect Neurosci. 2019 Apr 30;14(5):549–58. doi: 10.1093/scan/nsz032 (PMC6545532; doi:10.1093/scan/nsz032)

Supplementary Material: Structural MRI of patients

Patient 1

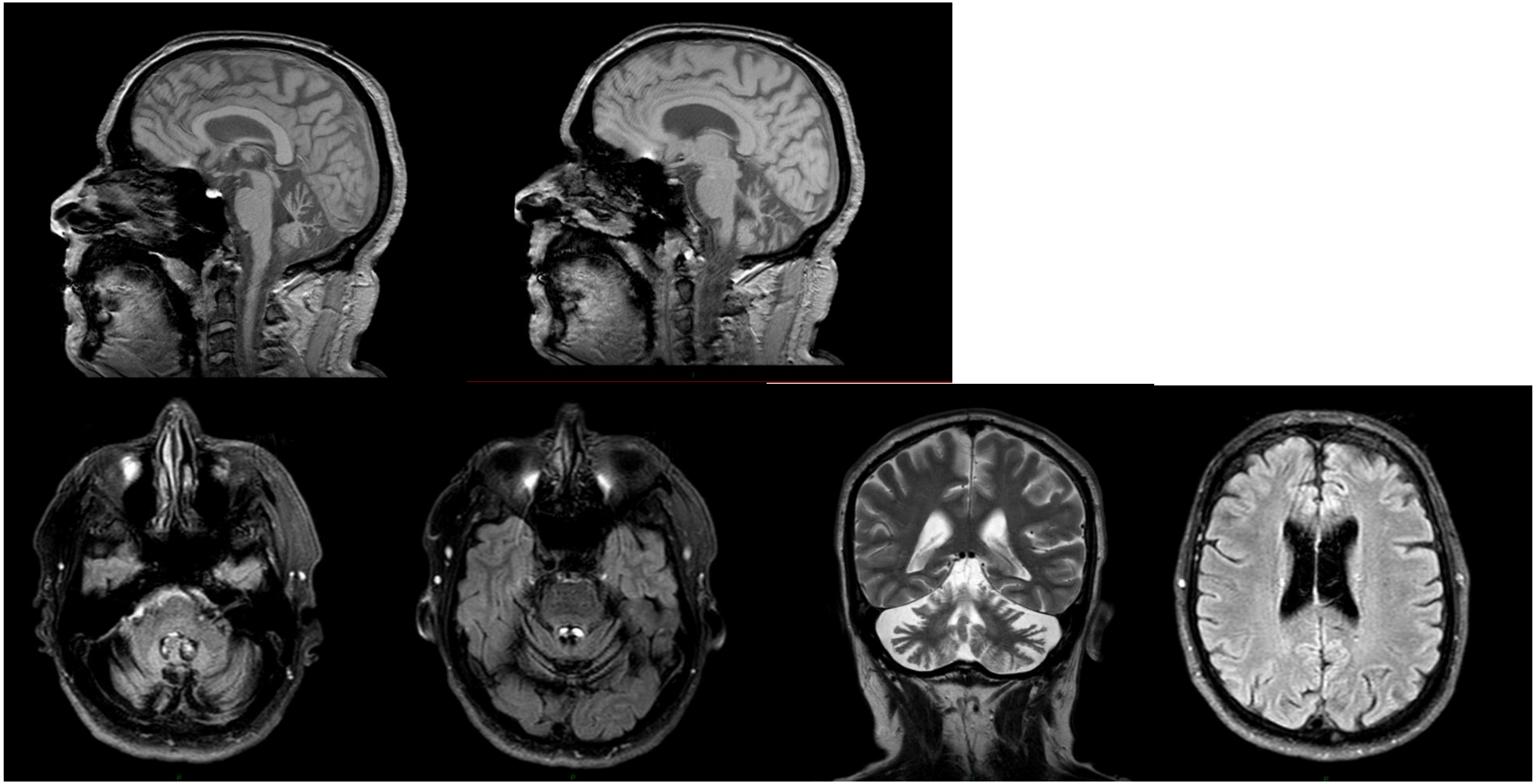

Patient 2

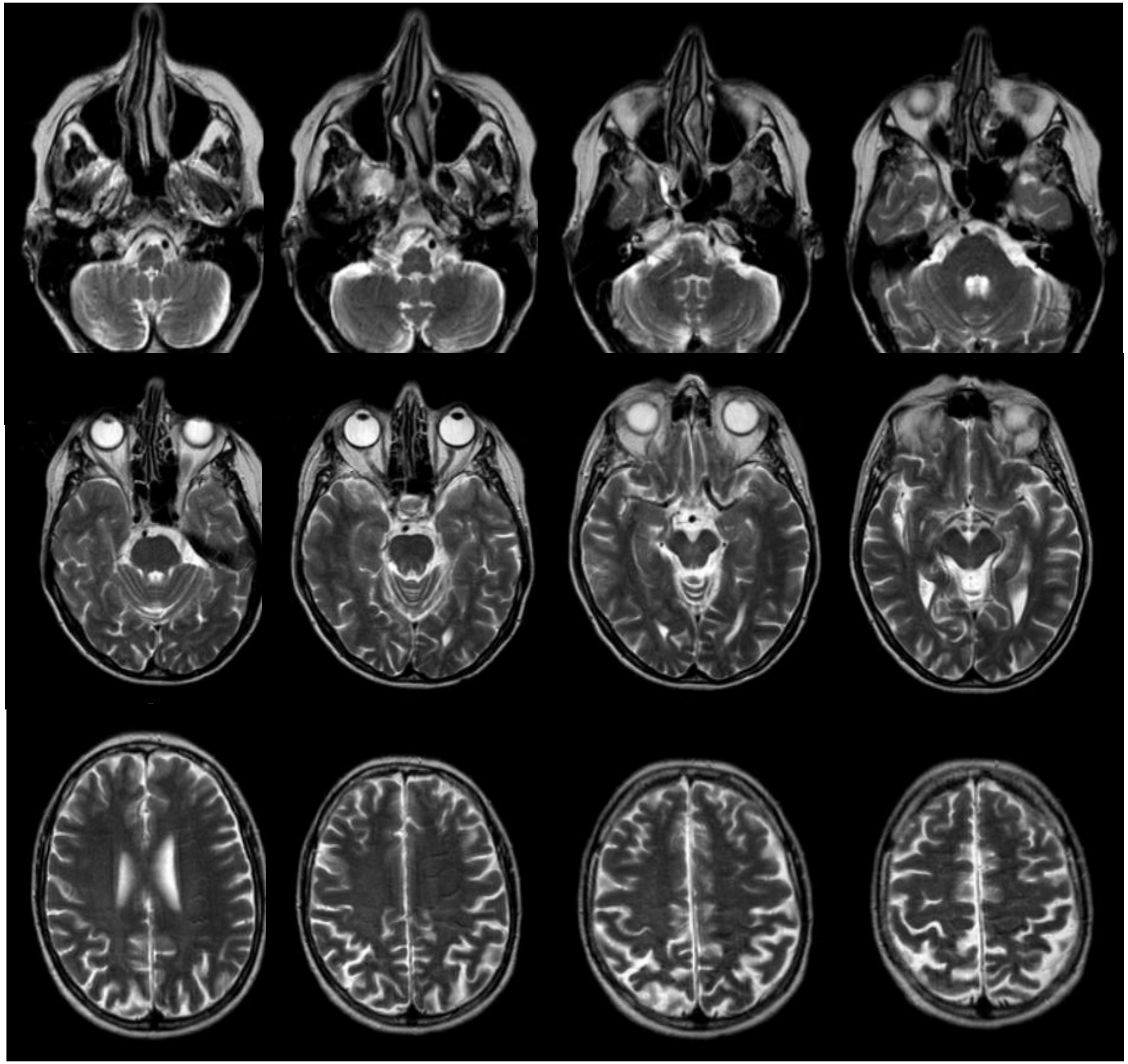

Patient 3

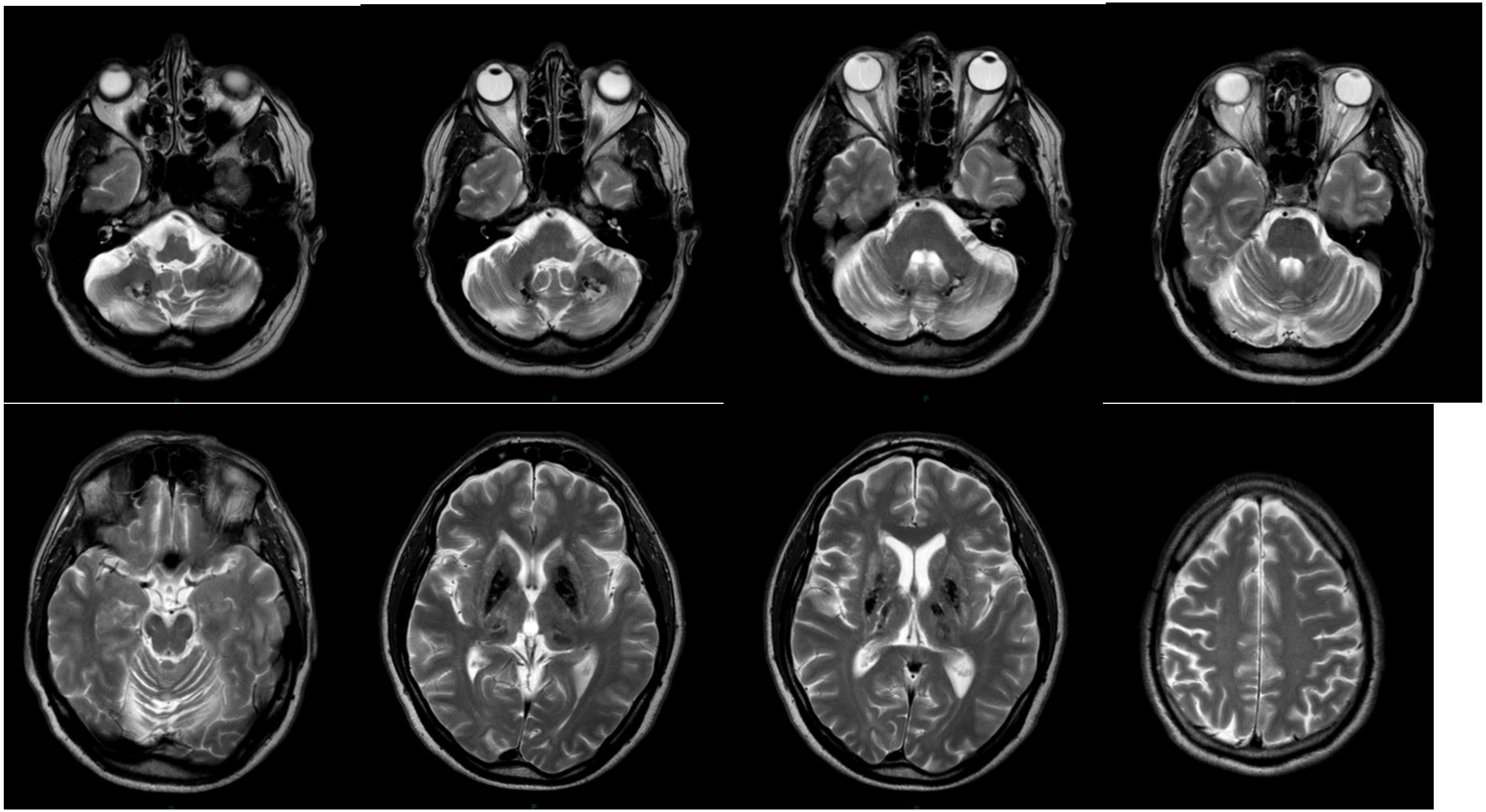

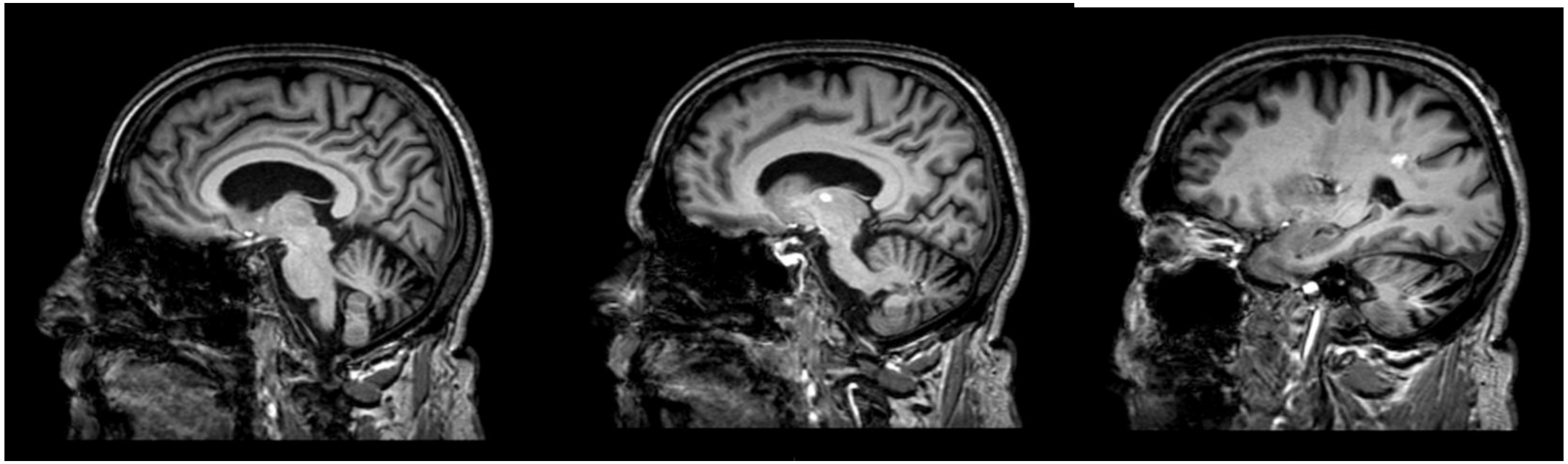

Patient 4

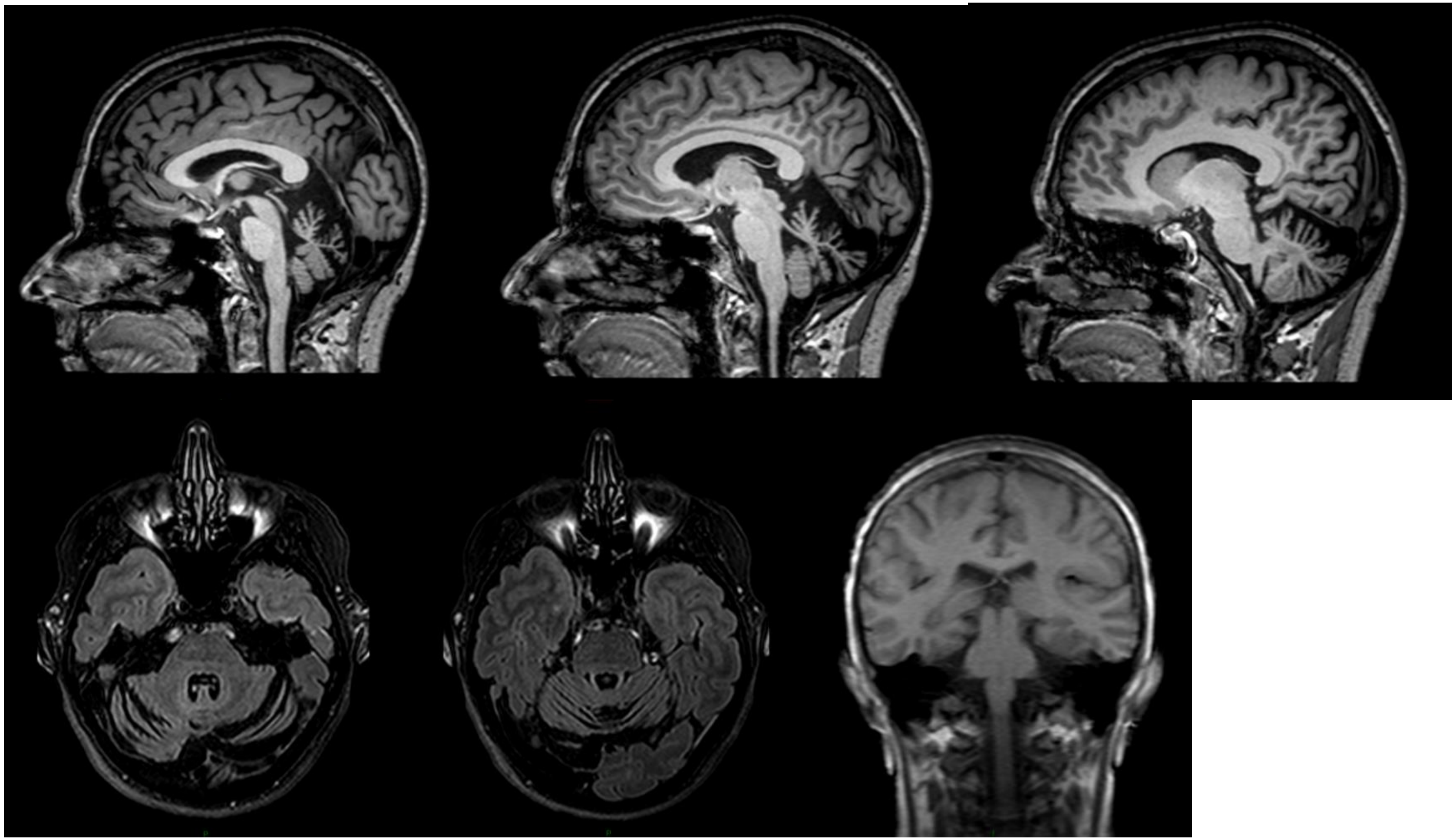

Patient 5

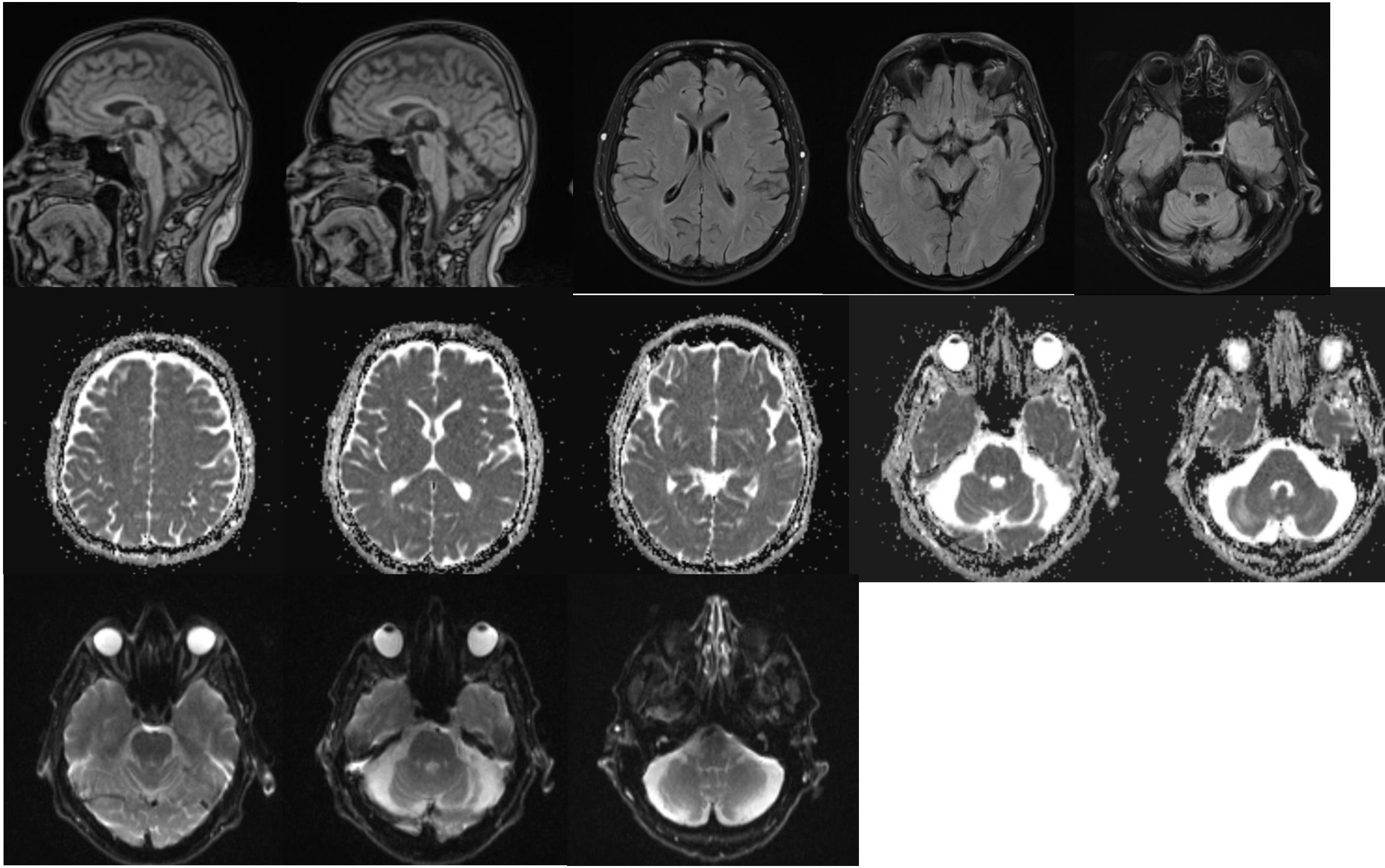

Patient 6

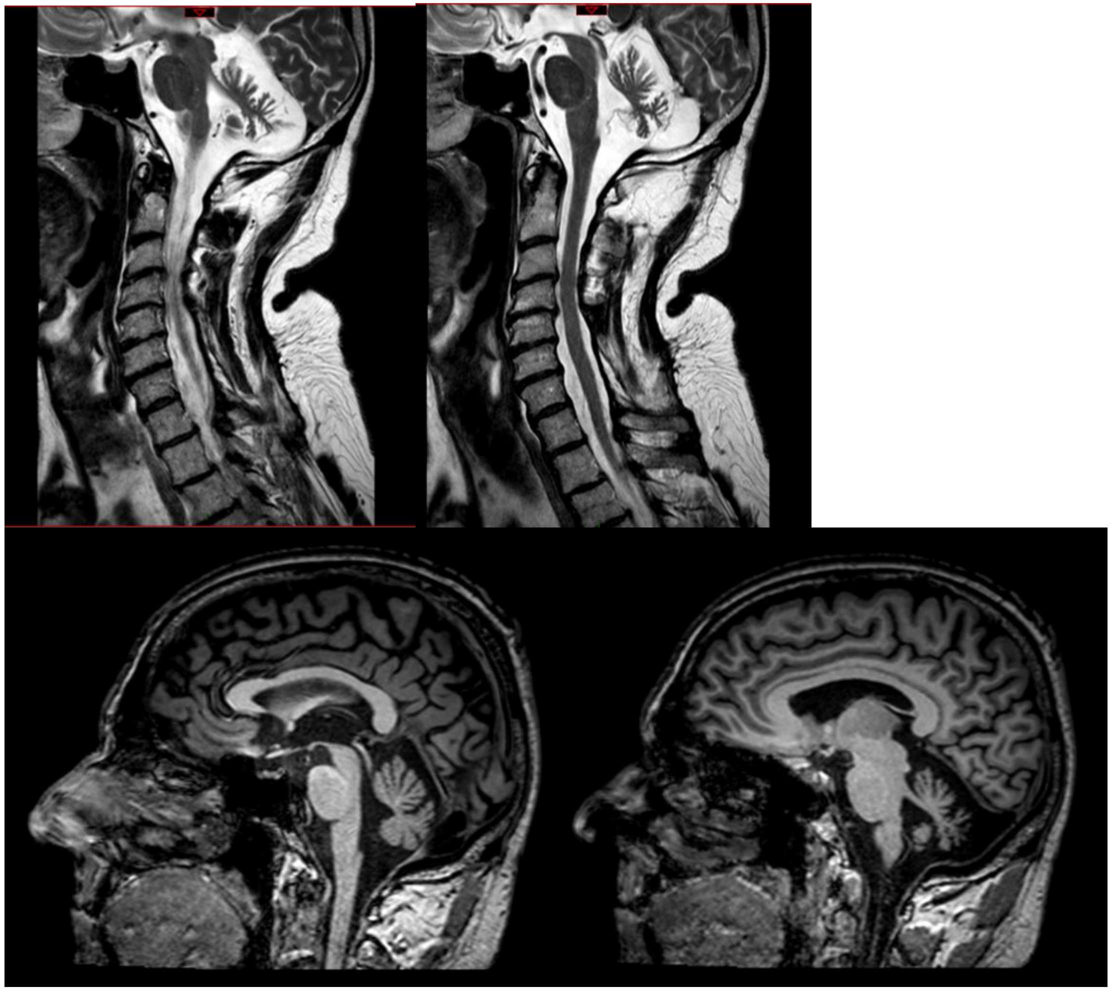

Patient 7

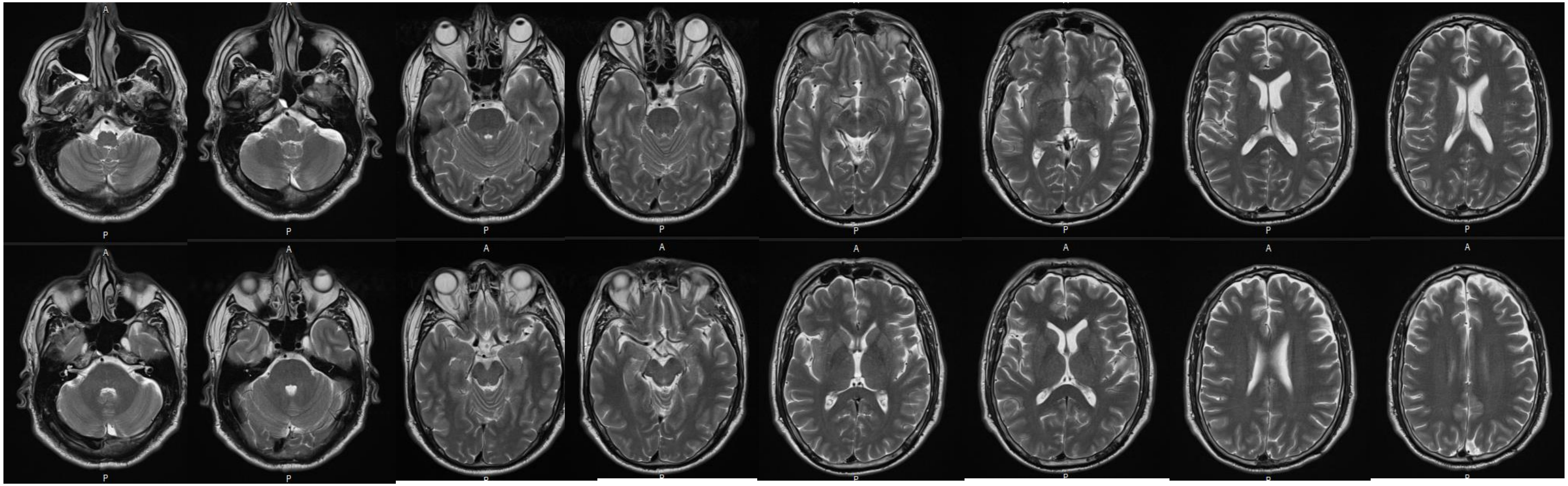

Patient 8

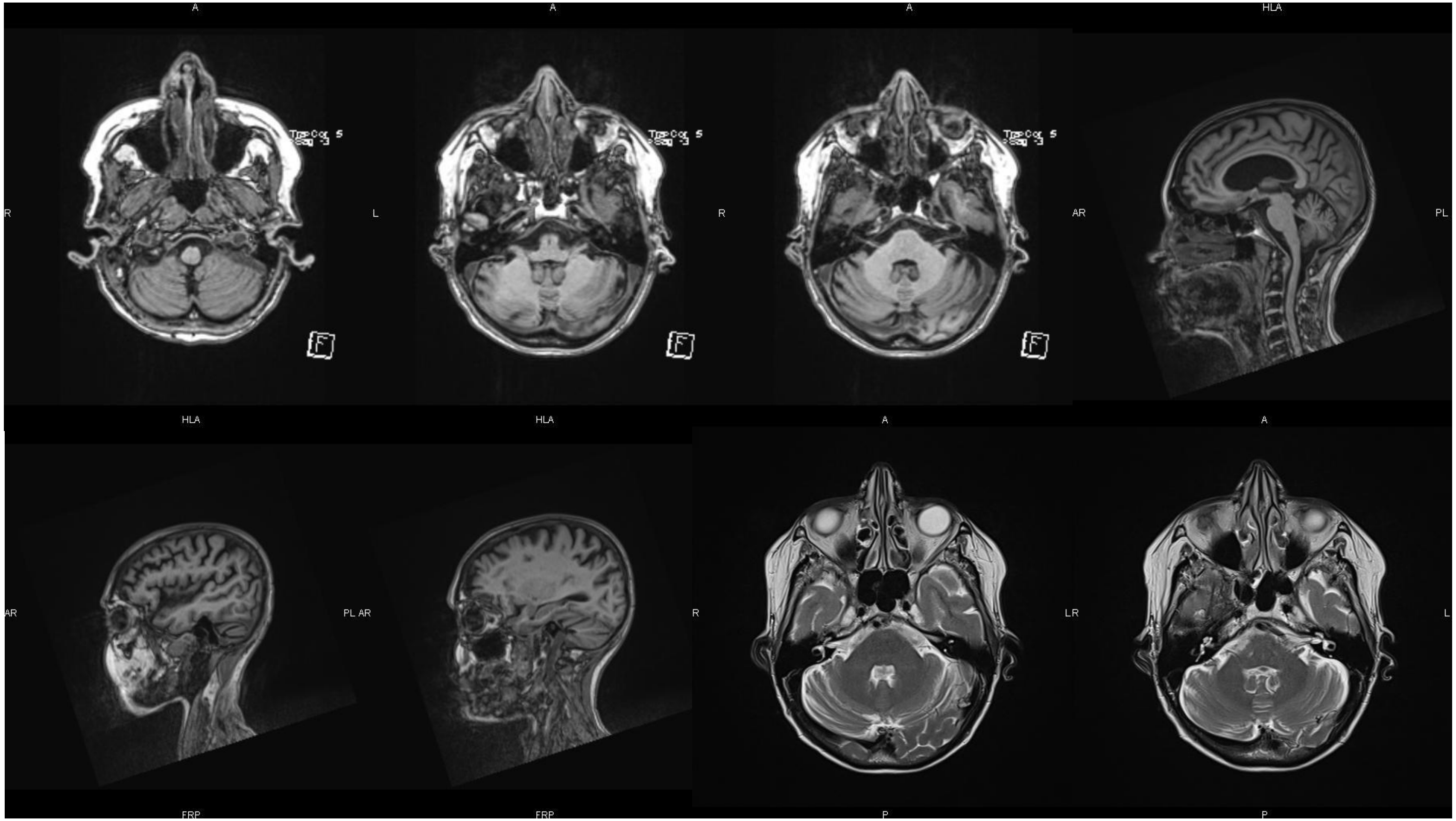

Patient 9

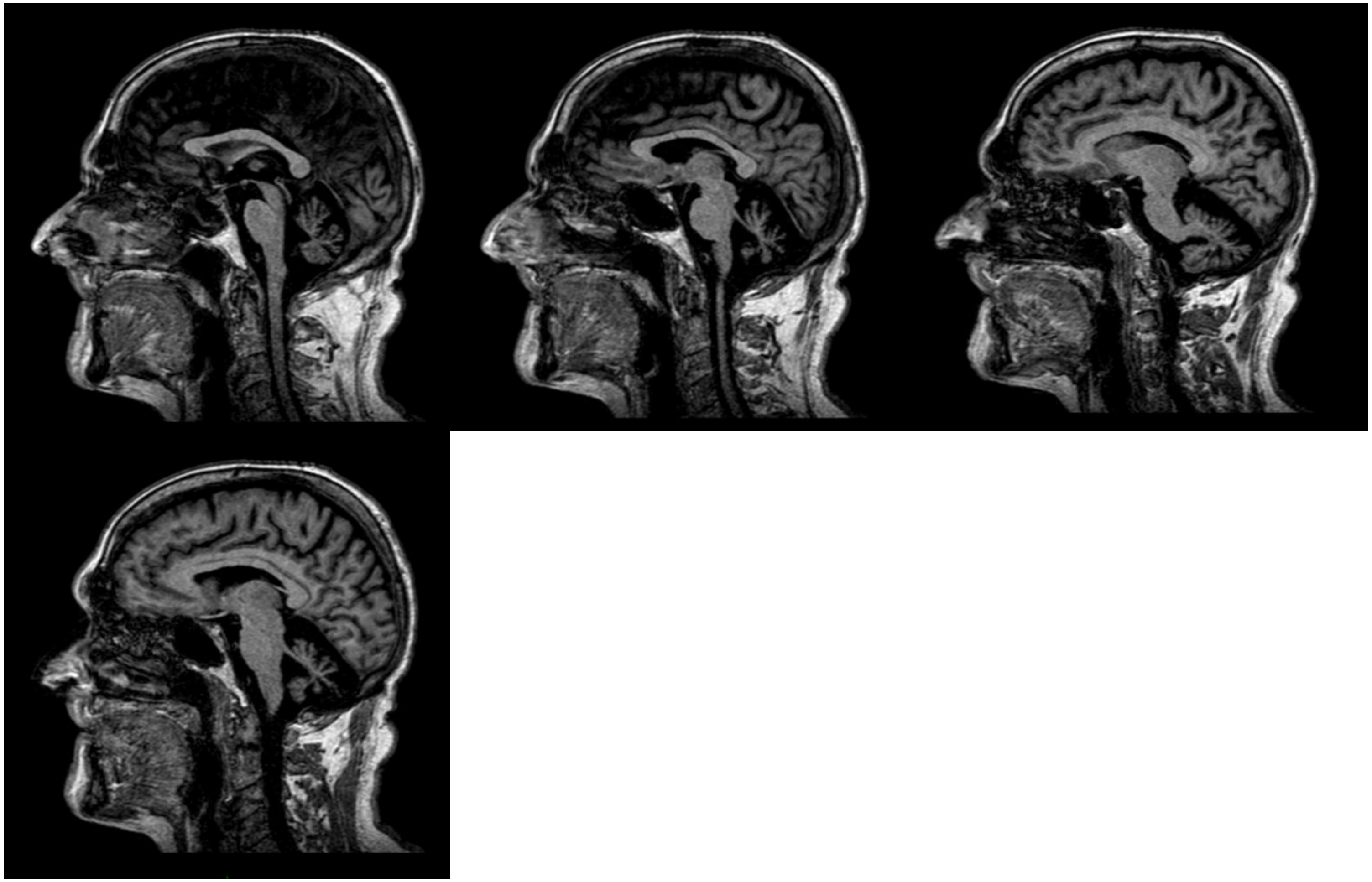

Patient 10

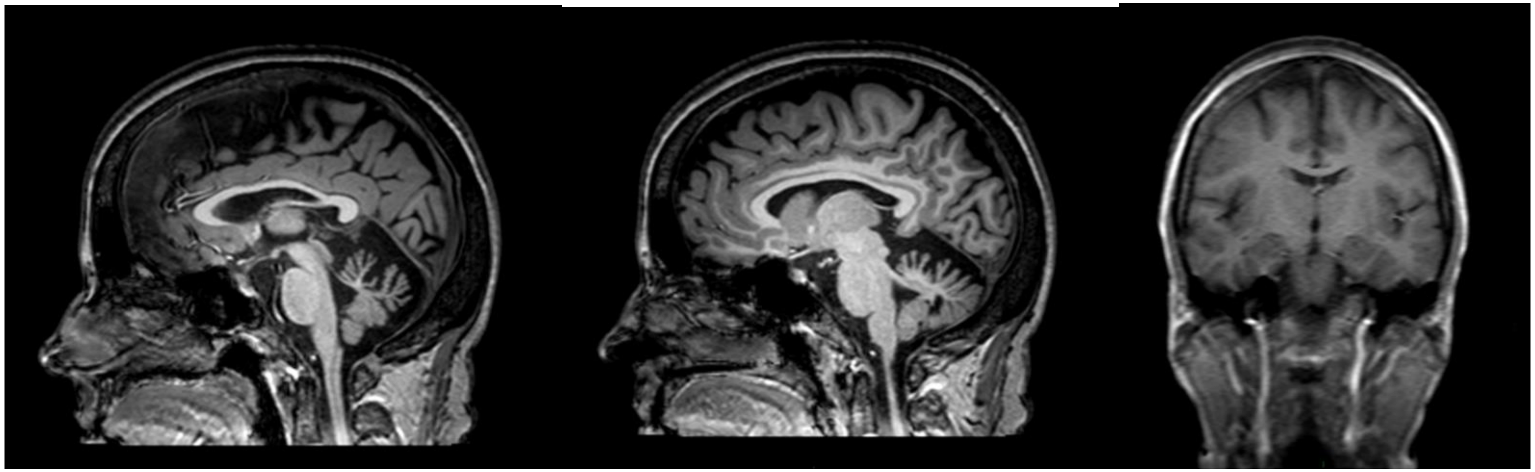

Patient 11

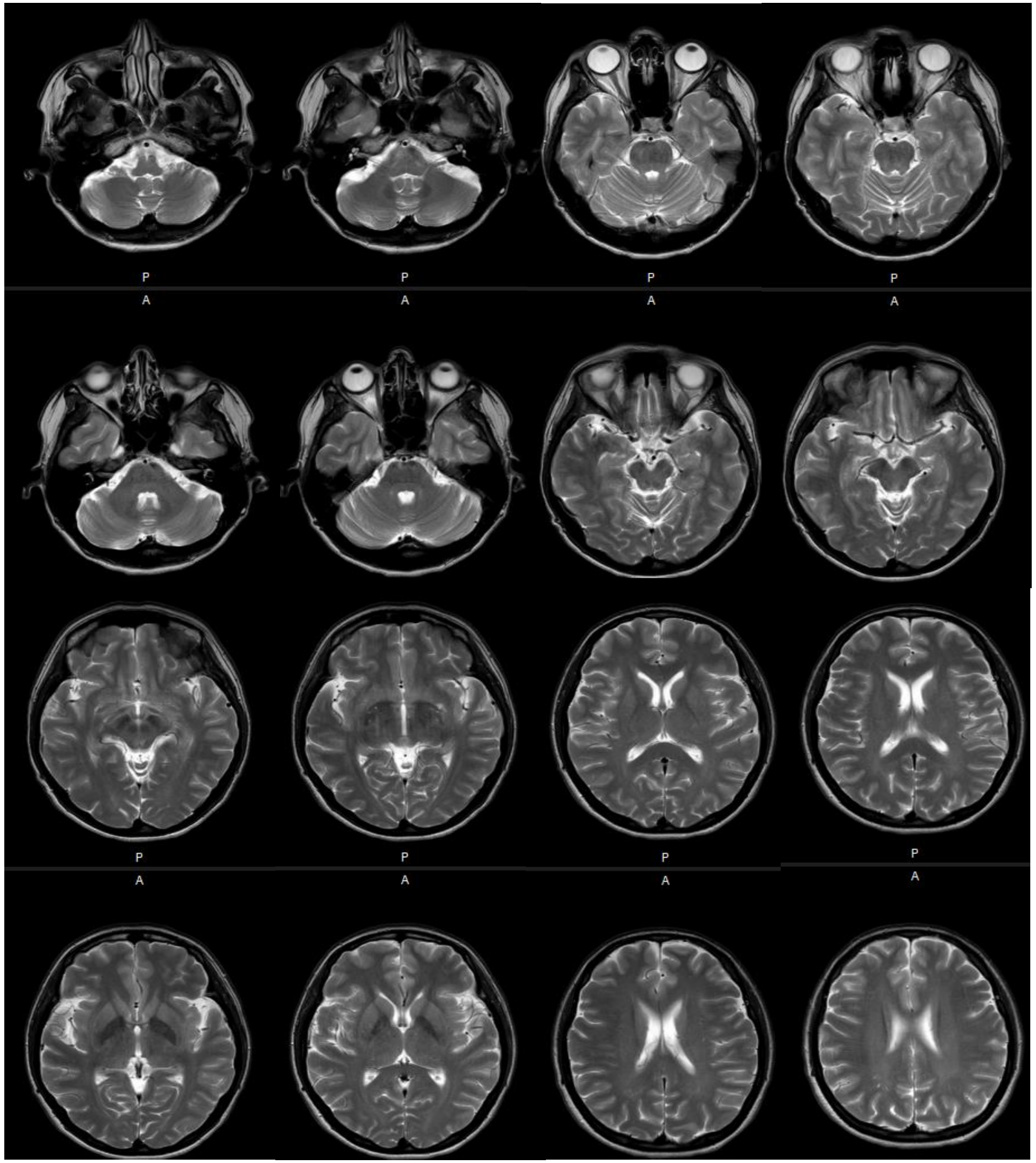

Supplement: scan-18-186-File002_nsz032 [file scan-18-186-file002_nsz032.pdf]
